# Temporal Discounting and Search Habits: Evidence for a Task-Dependent Relationship

**DOI:** 10.3389/fpsyg.2018.02102

**Published:** 2018-11-14

**Authors:** Mel W. Khaw, Ziang Li, Michael Woodford

**Affiliations:** ^1^Center for Cognitive Neuroscience, Duke Institute for Brain Sciences, Duke University, Durham, NC, United States; ^2^Department of Economics, Princeton University, Princeton, NJ, United States; ^3^Department of Economics, Columbia University, New York, NY, United States

**Keywords:** temporal discounting, eye movements, visual search, decision-making, heuristics

## Abstract

Recent experiments suggest that search direction causally affects the discounted valuation of delayed payoffs. Comparisons between options can increase individuals' patience toward future payoff options, while searching within options instead promotes impatient choices. We further test the robustness and specificity of this relationship using a novel choice task. Here individuals choose between pairs of delayed payoffs instead of single delayed outcomes. We observe a relationship between search styles and temporal discounting that are the opposite of those previously reported. *Integrators*—those who tend to compare attributes *within* alternatives—discount and choose more slowly than *comparators*—those who are more likely to compare *between* alternatives. This finding supports and augments the view that individuals' search strategy is predictive of subsequent discount rates. In particular, the direction of this relationship is further modifiable based on the spatial layout and varying information within an individual's decision-making environment.

## Introduction

Many theories of intertemporal choice (e.g., Samuelson, 1937; Laibson, 1997) assume that values should be assigned toward a decision-maker's options in a way that depends only on the various characteristics of each option. In such frameworks, an option's typical attributes—a monetary reward and an associated time delay (e.g., $5 in 15 days)—are integrated together in order to assign an overall utility or value to the option. Other models of time-delayed valuation (e.g., Read et al., 2013; Marzilli-Ericson et al., 2015) have instead proposed that decisions are made (either wholly or in part) on the basis of individual comparisons of particular attributes between available options. Recent experiments by Reeck et al. (2017) provide evidence that both types of cognitive processes—integration of the multiple attributes of a given option, and attribute-wise comparisons between options—play a role in value-based decision making, but to differing extents for different people. They find not only that subjects differ in the frequency with which they use different patterns of search while deciding, but that these differences in search also correlate with distinct choice patterns often attributed to individual preferences. Specifically, they find that integrative searchers—subjects with more frequent search transitions *within* an option—appear more impatient than comparative searchers—subjects with more frequent transitions *between* options.

This finding raises the question whether it means that information search strategy actually influences choice, or only that search strategy is for some reason correlated with the differing discount factors of different individuals. Reeck et al. (2017) address the issue of causality by exogenously manipulating subjects' search strategies; they find an effect of their causal manipulation, increasing either impatient or patient choices by impeding comparative or integrative transitions respectively. We provide further insight into the question of causality, complementing their investigation, by showing that a change in the organization of on-screen information can flip the sign of the correlation between visual search strategy and apparent discounting of future payments.

**Figure 1:**
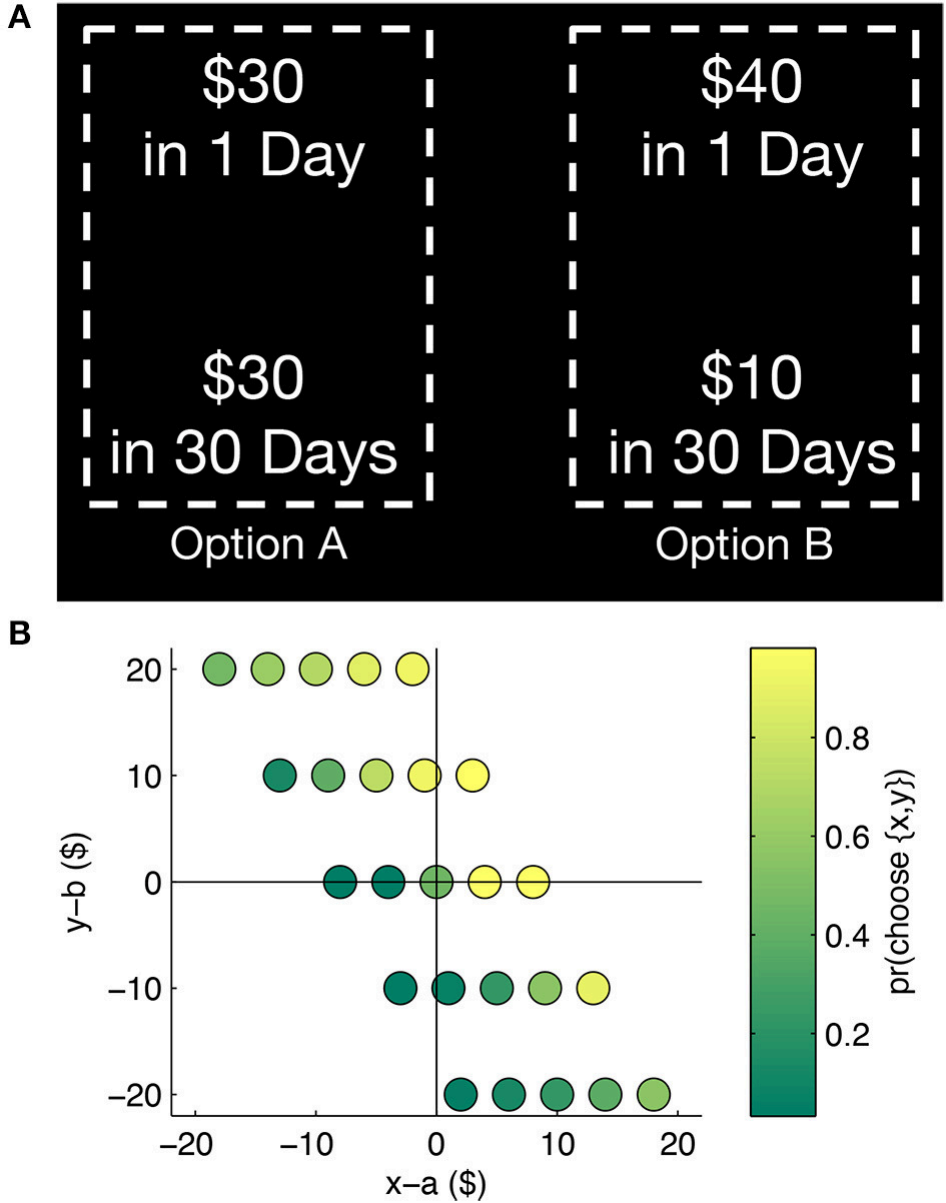
**(A)** Subjects made choices between pairs of delayed rewards while their eye movements were recorded. Attributes for each option would be revealed only when subjects' gaze was directed on a particular quadrant. Every option provides choices between alternatives {*x, y*} and {*a, b*}; in this example, *x* = 30, *y* = 30, *a* = 40, *b* = 10. **(B)** The payoff differences between attributes that were utilized in this study. The probability of choosing {*x, y*} increases as differences on both attributes increase toward the positive domain, indicating that subjects are responsive to increasing total payoffs.

We thus ask the following question: can we reverse the sign of the correlation between search styles and patience, simply by switching the informative roles of integrative/comparative search? In our task, subjects now choose between *pairs* of delayed rewards instead of single reward alternatives. Each option in our task promises a pair of variable monetary payoffs at the same “sooner” and “later” time points; only the payoff magnitudes belonging to each option varies across trials (Figure 1). In effect, the spatial positions delivering payoff and delay information in Reeck et al. (2017) now provide information about short-term and long-term payoffs respectively. We predict that comparative (integrative) transitions will now be associated with an increased level of impatience (patience). Previously, Reeck et al. (2017) suggest that comparative transitions drew attention toward differences in total payoffs between options, leading to increased patience. Meanwhile, integrative transitions in their task would display the delay and payoff of a single option, information that might emphasize time-discounted valuations (subsequently promoting impatience). In our task, total payoffs for a given option instead follow integrative transitions, potentially leading to an increased frequency of patient choices. Meanwhile, comparative transitions are now expected to emphasize short-term payoffs, if the previous interpretation regarding the increased weighting of payoff differences holds. Put another way, integrative and comparative transitions were associated with the increased weighting of delay and payoff information respectively; our task now replaces the spatial role of delay and payoff information with long and short-term outcomes. The current task thus pushes the link between search and discounting further; if search effects are mediated by the specific information brought forth by search, the sign of the original effect should reverse. We test this prediction by switching the temporal relevance of information supplied by each set of search strategies.

Similar to Reeck et al. (2017) and other studies of decision process tracing (Johnson et al., 1989), payoff and delay information were revealed only when subjects directed their gaze within the attribute's display region. We measured subjects' eye movements and choices as they acquired information and chose between delayed rewards. With these data, we re-examined the link between individuals' visual search and temporal discounting behavior.

## Methods

### Participants

Twenty-seven adults (15 female, age range = 18–32 years, *M* = 23.76, *SD* = 4.28) with normal eyesight completed the task on a computer and remote eye-tracking station in about 60 min. Participants were briefed about the details of the task and were compensated with a base fee of $10. All procedures were approved by the Columbia University Institutional Review Board under protocol #IRB-AAAQ2255.

### Apparatus

Experiments were conducted in a quiet, darkened, and isolated room, with an LED-backlit monitor (Dell P1914S; set at 1280 x 1024 resolution) positioned behind the eye tracker (Arrington Research MCU02 220 Hz monocular system) on a desk. Subjects performed the task on an ocular chinrest (Richmond Products Inc.) installed on the edge of the desk, 28.5 cm ahead of the monitor. Subjects performed the task with their chin resting comfortably in a chin cup and their foreheads resting against a cushioned forehead restraint. The height of the monitor was adjusted in order to place the center of the screen at approximately eye level. The observer sat facing the display, with their hand situated on the relevant keys on the keyboard. Keyboard inputs (left and right arrow keys) to the computer initiated the start of the task and also signaled the subjects' decisions. Each attribute's square bounding box subtended 14.25 degrees of visual angle. Vertical height between an option's attributes subtended 3.58 degrees of visual angle, while horizontal and diagonal distances between attributes of different options subtended approximately 17.06 and 17.42 degrees of visual angle respectively. Subjects were required to re-center their gaze at the center of the screen in order to initiate each trial. Attributes were displayed as soon as the recorded gaze was within the bounding box of each coordinate. The onset of attributes' display were thus constrained only by the eye movement recording rate (220 MHz) and the monitor refresh rate (60 MHz)—no lag or difficulty in revealing attributes were reported by subjects while performing the task.

### Procedure

Subjects were presented with a series of choices (200 in total) in which each option promised two rewards: one that was occurring sooner in time and one occurring at a later date (Figure 1A). Payoff information was presented at four quadrants of the screen; however, payoff or delay information in each quadrant was only revealed when subjects' gaze was directed within the rectangular bounding box of each attribute. In comparison to the task layout of Reeck et al. (2017), the top row of attributes correspond to “sooner” rewards rather than the monetary amounts of individual alternatives. Similarly, the bottom row of attributes now contain information about “later” rewards instead of the delay duration of individual alternatives. Subjects were not limited in the amount of time required to produce a decision. Before the initiation of each trial, subjects were required to align their gaze at a fixation cross positioned at the center of the display. The fixation cross was displayed for at least 1 s before subjects re-centered their gaze. The outline of the chosen option, along with the payoff information contained within, were displayed for 1 s upon the detection of a key press.

The delay associated with each payoff pair was held constant but the payoff magnitudes associated with each choice varied across trials. For instance, option A may promise $X in 1 day along with $Y in 30 days; option B then provides $A in 1 day and with $B in 30 days. In order to introduce trade-offs between total (long-term) and sooner payoffs, we designed the trials such that on the majority of the trials (64%), the inequalities *X* < *A* but *X* + *Y* > *A* + *B* (and vice versa) would hold. Patient behavior is thus defined and measured as choices that favor total payoffs at the cost of a reduction in sooner payoffs. The differences in payoffs at both timepoints ranged between –$20 and +$20, with the full range of differences specified in Figure 1B. Variation in the delay length was introduced between subjects. Subjects were randomly assigned to conditions in which later rewards occurred 30, 50, 100, or 150 days after participation. Five subjects were assigned to each condition except in the case of the 150 days delay; 12 subjects were assigned to that condition in order to increase the likelihood of observing impatient choices. Using the sub-population assignments described below (based on a median split performed on all 27 subjects), 3 integrators were identified in each delay condition barring the 150 days condition, in which 4 integrators were identified. A one-way between subjects ANOVA was conducted to determine if there were unintended treatment effects on the frequency of identified integrators/comparators. There were no significant differences in set membership between treatment conditions [*F*_(3, 23)_ = 0.58, *p* = 0.63]. The sooner payoff always occurred a day after the experiment. One randomly chosen trial was actualized for each subject's payment.

## Results

### Search strategy and choice

To summarize search strategy on each trial, we computed subjects' Payne Index (PI; Payne, 1976) as the relative difference between alternative and attribute-based transitions:

(1) $$\begin{array}{r} PI=\frac{Alternative-Attribute}{Alternative+Attribute} \end{array}$$

**Figure 2:**
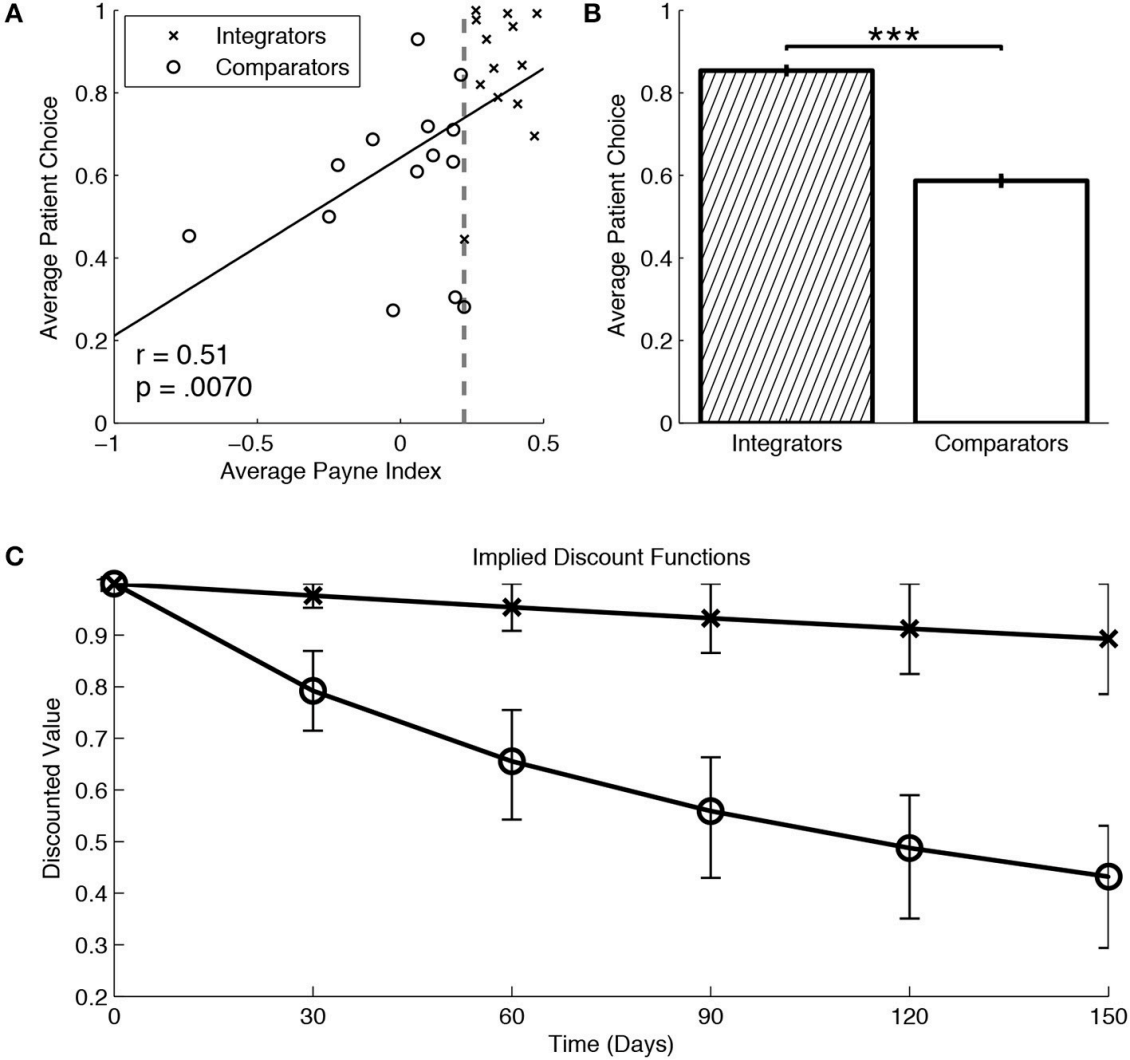
**(A)** Subjects' aggregate search strategy is correlated with the fraction of patient choices individually made; a median split (gray line) was used to identify integrators (crosses) and comparators (circles). **(B)** Integrators exhibit significantly higher number of patient choices compared to comparators. **(C)** The choices of integrators imply shallower discounting across time (relative to comparators) using the best-fitting parameters of a hyperbolic discount function (Mazur, 1987). All error bars denote the standard errors of their respective means (^***^ denotes significance at *p* < 0.001 level).

**Figure 3:**
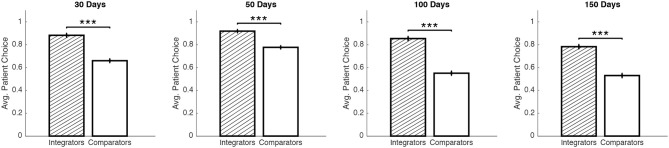
The difference in patient decision-making is also true for each subset of comparators and integrators in our study. Each panel displays the average frequency of patient choices made by members of sub-populations assigned in Figure 2A; the title of each panel denotes the delay duration of the second payoff throughout the task (^***^ denotes significance at *p* < 0.001 level).

PI was reliably measured within subjects (Cronbach's alpha = 0.99). We then identified integrative and comparative searchers using a median split of our pooled sample across delays based on subjects' average PI. The median split offers a simple algorithm-free method of identifying potentially different sub-populations in our data; additionally, using the mean (0.17) instead of the median (0.22) does not alter set membership or subsequent comparisons significantly. Alternatively, a K-means clustering algorithm (performed on average PI and patient choice frequency, similar to Reeck et al., 2017), also suggests for a 2-cluster solution with set membership that closely matches the median split. An individual's average PI was highly correlated with the fraction of patient choices made during the task (Figure 2A), *r*_(25)_ = 0.51, *p* < 0.01. Based on our median split, integrators are associated with a greater proportion of patient choices (Figure 2B), χ^2^(1, *N* = 3456) = 302.01, *p* < 0.001. Furthermore, we verify that this relation isn't driven by particular delay duration conditions in our study (recall that delays were held constant for each subject in our study). Proportion tests between members of the two sub-populations in each delay treatment reveals a significant difference in patient choices for each delay (Figure 3): 25 days [χ^2^(1, *N* = 640) = 46.51, *p* < 0.001], 50 days [χ^2^ (1, *N* = 640) = 26.10, *p* < 0.001], 100 days [χ^2^ (1, *N* = 640) = 72.20, *p* < 0.001], and 150 days [χ^2^ (1, *N* = 1536) = 92.18, *p* < 0.001]. Returning back to the pooled data, choices of comparators also imply a steeper average discount rate by a factor of 10 (Figure 2C), using the hyperbolic discount function proposed by Mazur (1987). A saccade identification analysis confirms the greater relative density of vertical vs. horizontal saccadic transitions for integrators vs. comparators (Figure S3).

### Psychometric curves

**Figure 4:**
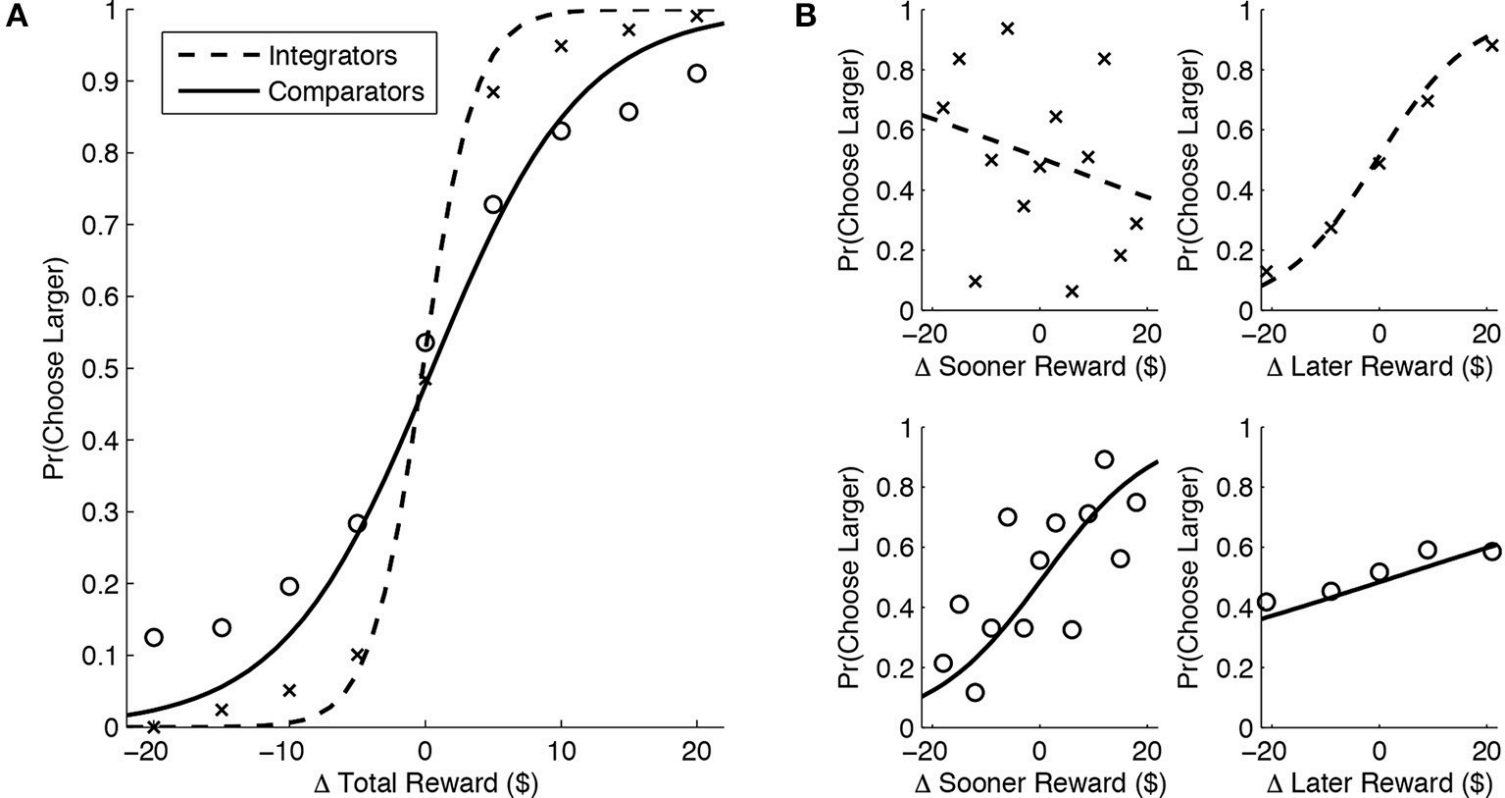
**(A)** Integrators choices are more driven by the total value difference relative to comparators, as indicated by the larger degree of steepness in their average psychometric functions. **(B)** Integrators' choices (top row) are sensitive primarily to the delayed payoff differences, while comparators decisions (bottom row) are driven mostly by differences in payoffs that occur sooner, with an intermediate sensitivity to the later reward.

In order to examine sensitivity toward intra- and inter-attribute payoff differences, we analyzed subjects' choices as a function of payoff differences occurring at different timeframes. To achieve this, we modeled psychometric choice functions using basic 2-parameter sigmoid functions of the form:

(2) $$\begin{array}{r} Pr\left(Choice\right)=\frac{1}{1+e^{-a\left(\Delta \$-b\right)}} \end{array}$$

where Δ$ refers to the difference in payoffs at either the sooner, later, or overall timeframe; in addition, *a* and *b* are free parameters that dictate the slope and intercept respectively for the estimated choice functions. The parameter *a* can thus be interpreted as subjects' *sensitivity* toward differences in payoffs while *b* represents the estimated value difference that would make the subject indifferent between either alternative. Choice functions were fitted to observed behavior using maximum likelihood estimation—parameters were chosen to maximize the log likelihood of observing the subjects' choices. The fitted parameters for choice functions belonging to each individual highlight several further differences between sub-populations. Firstly, integrators exhibit a greater average sensitivity toward total payoffs independent of delay duration, *t*_(25)_ = 3.83, *p* < 0.001 (Figure 4A). In addition, integrators are more sensitive to the *later* payoff differences between the two options, *t*_(25)_ = 4.20, *p* < 0.001, and less sensitive to differences at shorter delays, *t*_(25)_ = −3.10, *p* < 0.01 (Figure 4B).

### Search strategy and choices over time

Following a reviewer's suggestion, we additionally tested for differences in choice behavior as a function of time. We examined for changes in three forms of decision-related behavior over time: (i) decision time, (ii) subjects' Payne Indices measured at the level of individual trials, and (iii) subjects' likelihood of producing a patient choice (as defined earlier). Following previous studies of adaptive performance and learning effects over time, e.g., Thach et al. (1992) and Martin et al. (1996), we summarize observed dynamics (or a lack thereof) using simple exponential decay/growth functions:

(3) $$\begin{array}{r} f\left(x\right)=a\left[e^{bt}\right] \end{array}$$

**Figure 5:**
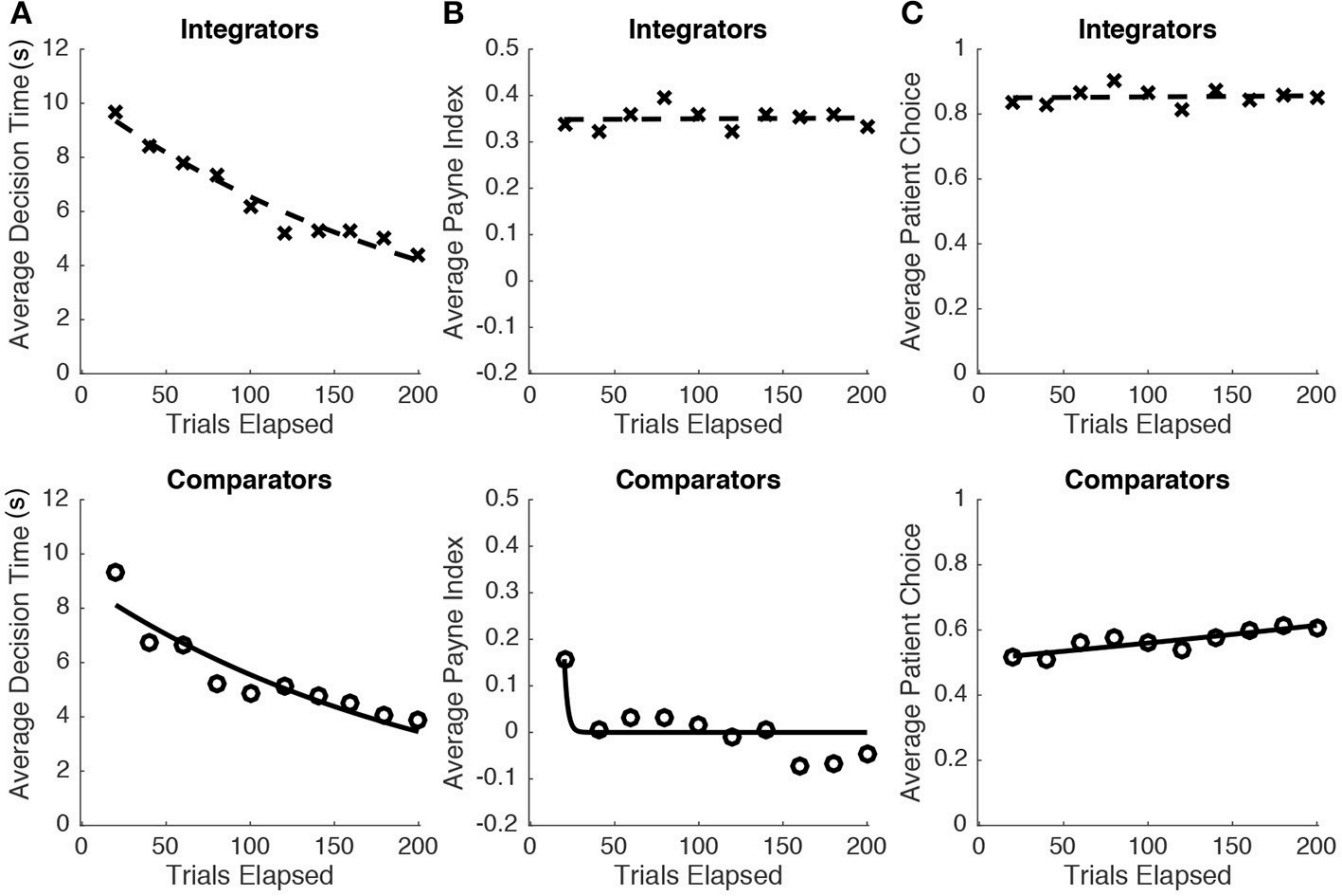
**(A)** For both integrators and comparators, decision time decreases constantly throughout the task, suggesting that subjects are becoming more efficient in producing decisions over time. **(B)** The changes in decision time are accompanied by a decrease the average Payne Indices of comparators (making comparative transitions even more likely by the end of the task); the search strategies employed by integrators are constant throughout the task. **(C)** The probability of choosing the patient option was stable over time within both sub-populations.

Where *a* determines the starting point of the function (e.g., average decision time at *t*_0_, the beginning of the task) and *b* determines the rate of exponential growth/decay over some time *t*. Group and individual measures were obtained by averaging across 20 trial blocks (out of 200 total trials). The resulting parameters were obtained by iteratively minimizing least squared errors (Marquardt, 1963) from averaged values at each timepoint. To test for significant changes in average choice-related behaviors over time, we test whether the parameter *b*, fit over single individuals, differs significantly from 0. A significant non-zero value of *b* would suggest some form of decay/growth trend over time. We find that reaction time decreases over the course of the task for both integrators [*t*_(12)_ = −7.15, *p* < 0.001] and comparators [*t*_(13)_ = −8.21, *p* < 0.001]. Payne Indices do not shift over time for integrators [*t*_(12)_ = 0.14, *p* = 0.89], though it does decrease over time for comparators [*t*_(13)_ = −2.16, *p* < 0.05]. Critically, choice behavior is stable over time for both integrators [*t*_(12)_ = 0.14), *p* = 0.89] and comparators [*t*_(13)_ = 1.93, *p* = 0.080]. To visualize these similarities and differences between groups, we fit these functions toward pooled averages (across groups of *N* = 13 and *N* = 14 for integrators and comparators respectively). Figure 5 and Table 1 shows the fitted parameters of the resulting exponential functions to the pooled data.

**Table 1:** Parameter estimates for population-level exponential growth/decay curves.

**Variable**	**Integrators**				**Comparators**			
	***a***	**95% C.I**.	***b***	**95% C.I**.	***a***	**95% C.I**.	***b***	**95% C.I**.
Decision time	10.14	(9.21, 11.08)	–0.0046	(–0.0056, –0.0036)	8.85	(7.73, 9.98)	–0.0049	(–0.0063, –0.0035)
Payne index	0.35	(0.32, 0.38)	0.00060	(–0.00069, 0.00081)	0.23	(–0.23, 1.56)	–0.10	(–0.21, 0.0081)
Patient choice	0.84	(0.80, 0.88)	0.00014	(–0.00040, 0.00043)	0.52	(0.47, 0.57)	0.0012	(0.00044, 0.0020)

### Within-subject regression analysis

We further examine how trial-by-trial variation in payoffs and search strategy affect the choices of each individual subject. Given previous results demonstrating that impeding particular transitions alters subsequent choices in a causal manner (Reeck et al., 2017), within-subject variation in search might also explain subsequent choices on a trial-by-trial basis. To this end, we conduct a logistic regression on each individual's data to explain the probability of producing a patient choice. As before, patient choices are defined as choices in favor of the option that provides greater total payoffs, while providing a smaller payoff at the sooner timepoint. 64% of trials for each subject involved the opportunity to make a trade-off of this kind. Given the limited number of trials (128) for each subject, we limit our predictor variables toward main factors known to be predictive of choice. We include the following as independent variables in our regressions: payoff magnitudes, gaze percentage as a measure of relative attention (Armel et al., 2008; Krajbich et al., 2010), as well as search strategy. The resulting regression formula was:

(4) $$\begin{array}{r} Pr\left(PatientChoice\right)=\frac{1}{1+e^{-\left(\beta_{0}+\beta_{1}{\$}_{p}+\beta_{2}{\$}_{i}+\beta_{3}G_{p}+\beta_{4}G_{i}+\beta_{5}PI+\epsilon \right)}} \end{array}$$

Where $ is the payoff magnitude belonging to either the patient or impatient option; *G* is the percent gaze recorded on the patient or impatient option; and, *PI* are subjects' Payne Indices computed on the basis of transition counts on each trial^1^. Since delays are held constant for each individual, we omit this influence from this individual-level analysis. The regression equation was fit to each individual subject by maximizing the log likelihood of observing an individual's choices. The estimated regression coefficients were then examined for significant differences using bootstrapped one-sample t-statistics. The average regression weights indicate significant influences of payoff magnitudes, as well as gaze directed to the payoff option, confirming the set of known influences on choice (Table 2). However, the trial-by-trial indices of search strategy were not a significant predictor of patient choices.

**Table 2:** Logistic regression estimates, along with bootstrapped confidence intervals and significance tests, of payoff, gaze, and search variables on patient choice frequency.

**Predictor**	**β**	**S.D**.	**95% C.I**.	***p*-value**
Payoffs (Patient)	0.33	0.33	(0.21, 0.46)	<0.001
Payoffs (Impatient)	–0.35	0.33	(–0.48, –0.24)	<0.001
Gaze % (Patient)	0.030	0.045	(0.015, 0.048)	<0.01
Gaze % (Impatient)	–0.058	0.14	(–0.12, –0.019)	0.28
Payne index	–0.0041	0.01	(–0.0093, 0.0009)	0.14
(Constant)	6.22	22.73		

## Discussion

Our results confirm and replicate the finding that individual differences in search habits are related to temporally discounted valuations. Furthermore, the direction of this association is additionally modifiable based on the type of information received; given a fixed dichotomy of search stragies (comparative or integrative search), such patterns are now linked in the opposite manner to patience than in previous experiments (Reeck et al., 2017). The directionality of these effects might depend on the task-specific implementation of specific decision heuristics. For example, in the present study, individuals concerned with the total payoffs of each offer would have to acquire information in a column-wise direction—consistent with the definition of integrative transitions. In the task used by Reeck et al. (2017), comparative transitions might instead be more useful for identifying the larger reward; in that case, comparative transitions between each payoff offer would suffice in comparing total rewards. Thus, search patterns characteristic of patient and impatient decision-makers might be better understood by their efficacy in allowing the observer to determine the most rewarding long-term and short-term outcomes. In our task, subjects' deployment of such strategies appear stable (Figures 5B,C) such that inter-trial variation in search and choice are not significantly related (Table 1). Further research on within-subject variability might explore causal manipulations, such as the time-lags introduced by Reeck, Wall and Johnson (2017), or the lengthening of gaze travel distances. Such manipulations would increase variability in search, beyond the range occurring naturally within subjects. Regardless, we demonstrate that the association between search and choice is robust enough, that one can effectively predict the sign of this relation based on the arrangement of on-screen information.

The present study also raises other general insights regarding search and choice behavior. Several control analyses are able to rule out other search-based interpretations of our results. First, we confirm that the percentage of gaze time distributed between spatial quadrants are approximately even for both sub-populations; as such, different groups are not choosing to simply ignore particular information (Figure S1A). Upon a reviewer's suggestion, we also examine the prevalence of search habits consistent with common reading directions. For example, decision-makers might be inclined to survey the attributes in Figure 1A clockwise from top-left to top-right, followed by counter-clockwise from bottom left. Examining the first set of transitions on each trial reveals that both sub-populations engage in such a search (Table S1). This transition pattern accounts for about 10% of the first four attributes viewed in both comparators and integrators. Although this pattern cannot explain average differences in patience here, the role of automatic search tendencies across different tasks is an important topic for future study. Further experiments are also warranted to determine whether choices are directly influenced by the sequence of information acquisition (in which case one might consider delivering individuals fixed patterns of information), or are instead reflective of participants' chosen strategies in this task (which are related to their choices). Given the single study here, we also cannot disambiguate between a stable family of search strategies that would be consistent across tasks, and correlations between choices and search that manifest differently in a random manner across tasks. Our data does suggest that the implementation of strategies are relatively stable during the length of the experiment (Figure 5). Nevertheless, the collective results now refute the view that choices are independent of factors beyond the presentation of complete information—choices are tightly related to the sequence of search as well as the type of information that is being received. In order to fully understand the range of search effects on choice, future work should study choices made by the same individuals across different tasks—ranging from traditional binary choice experiments (with no restrictions on sequential search), to paradigms that promote or even necessitate particular search patterns.

These results also call to attention the possible relevance of singular transition types outside of the Payne Index framework. For instance, the comparator/integrator distinction is ambiguous with respect to possible distinctions between horizontal vs. diagonal search. Diagonal transitions are classified as comparative in the current analysis, in order to aid comparisons with the results of Reeck et al. (2017), who find that diagonal transitions from payoff to delay are characteristic of comparators. Spatially-equivalent diagonal transitions indeed account for 55% of all diagonal transitions in our data. Beyond this study, diagonal transitions might also be interpreted as neither intra-attribute nor intra-alternative—they have been previously considered as a marker for the beginning of new search processes (Payne and Braunstein, 1978). In our data, the difference between comparators and integrators are more pronounced in the horizontal transition category (Figure S2A), though comparators perform more of both types of transitions. Although the omission of diagonal transitions does not change our main conclusions (Figures S2B,C), the role of such theoretically ambiguous transitions remains an interesting aspect for future experiments. With regards to other types of transitions, individual transition counts (summarized as Payne Indices per trial, or separately as horizontal, vertical, and diagonal transition counts) are not predictive of choices on a trial-by-trial basis (Table 1). Although these finer distinctions were not particularly useful in further explaining behavior here, future work in this vein would benefit from finer grain distinctions beyond within-attribute and within-alternative search.

Nevertheless, despite its simplicity, the broad classification of comparators and integrators yields other significant differences between these sub-populations. The transition patterns map on to other major forms of decision dynamics, such as the distribution of identified saccades (Figure S3). Furthermore, we are able to confirm that integrators exhibit slower reaction times, perform saccades at slower velocities, and execute a greater number of identified saccades per trial (Figure S1B). These differences might reflect a greater cognitive load brought forth by integration, or a lower opportunity cost of decision time for integrators. The latter is consistent with the finding that impatient individuals produce more vigorous eye movements in similar choice tasks (Orquin and Loose, 2013; Choi et al., 2014). Future studies might examine in further detail how these other dynamics relate to discounting; in particular whether these are also subject to change according to the task at hand. Indeed, comparators' quicker reaction times (Figure 5A, Figure S1B), observed variability in strategy over time (Figure 5B), and intermediate consideration of patient outcomes (Figures 4B, 5C) suggests that particular decision-making styles are inherently more variable across measures. Furthermore, the selection of decision heuristics might be made (in a meta-cognitive manner) to match specific cognitive limitations of the decision-maker (Cokely and Kelley, 2009). In this respect, search strategy offers a potential summary indicator of how decision-makers adapt in a particular task, while being related to a variety of other measures.

In summary, we replicate an apparent duality in aggregate search and discounting behavior, supporting the overall notion that the sequence of information one tends to receive is an effective predictor of intertemporal choice tendencies. In addition, we observe a relationship between search strategy and apparent time preferences that are the opposite of those previously reported—placing added emphasis on the type of information received, given a set of search patterns. Thus, insofar as particular search styles might promote increased consideration toward particular attributes, the directionality of these effects can be changed depending on the spatial layout and varying attributes of the task at hand. Overall, the robustness of such effects, independent of their directionality, encourages further work on process-level observations (Payne et al., 1988) and their relation to individuals' apparent time preferences.

## Author contributions

MK, ZL, and MW designed the study, analyzed the data, and wrote the paper. MK and ZL performed data collection.

### Conflict of interest statement

The authors declare that the research was conducted in the absence of any commercial or financial relationships that could be construed as a potential conflict of interest.

## Footnotes

^1^Individual transition counts are co-linear with PI as the latter summarizes individual transition frequencies. Replacing PI with individual transitions does not alter the conclusions here.
