# Supplementary material for: Temporal Discounting and Search Habits: Evidence for a Task-Dependent Relationship
Source: Front Psychol. 2018 Nov 14;9:2102. doi: 10.3389/fpsyg.2018.02102 (PMC6246652; doi:10.3389/fpsyg.2018.02102)
Supplement: Supplementary file 1 [file Data_Sheet_1.pdf]

# Supplemental Material for "Temporal discounting and search habits: evidence for a task-dependent relationship"

Mel W. Khaw
Duke Institute for Brain Sciences, Duke University
Ziang Li
Department of Economics, Princeton University
Michael Woodford
Department of Economics, Columbia University

## Saccade Analysis

To confirm differences in search strategy in terms of eye movements, we identified fixations and their associated saccades using a simple three-parameter spatial thresholding algorithm (Krassanakis, Filippakopoulou, & Nakos, 2014). Initial spatial thresholding was performed with a minimum cluster radius of 10 pixels within the $\{x, y\}$ recorded coordinate space and a minimum duration of 350 ms. Then, we classified vertical and horizontal saccades based on whether the movement covered more distance on the x or y-axis. Using this metric, we observe that integrators are associated with a greater frequency of vertical saccadic movements, while comparators perform more frequent horizontal saccades (Fig. 3).

## Initial Search Pattern Analysis

Following a reviewer's suggestion, we examined the initial search paths deployed by our subjects, generally and within the two identified sub-populations. This allows us to check for prior biases in search (such as natural reading directions). To do this, we recorded the sequences of visiting the first four quadrants on each trial, using subjects' recorded gaze coordinates. Table 1 displays a breakdown of occurrences of non-returning patterns only (omitting for brevity instances where subjects returned to a previously viewed attribute). The percentage of occurrences were computed based on the total number of observations attributed to each of the 1024 possible patterns.

## References

Krassanakis, V., Filippakopoulou, V., & Nakos, B. (2014). Eyemmv toolbox: An eye movement post-analysis tool based on a two-step spatial dispersion threshold for fixation identification. *Journal of Eye Movement Research*, *7*(1).

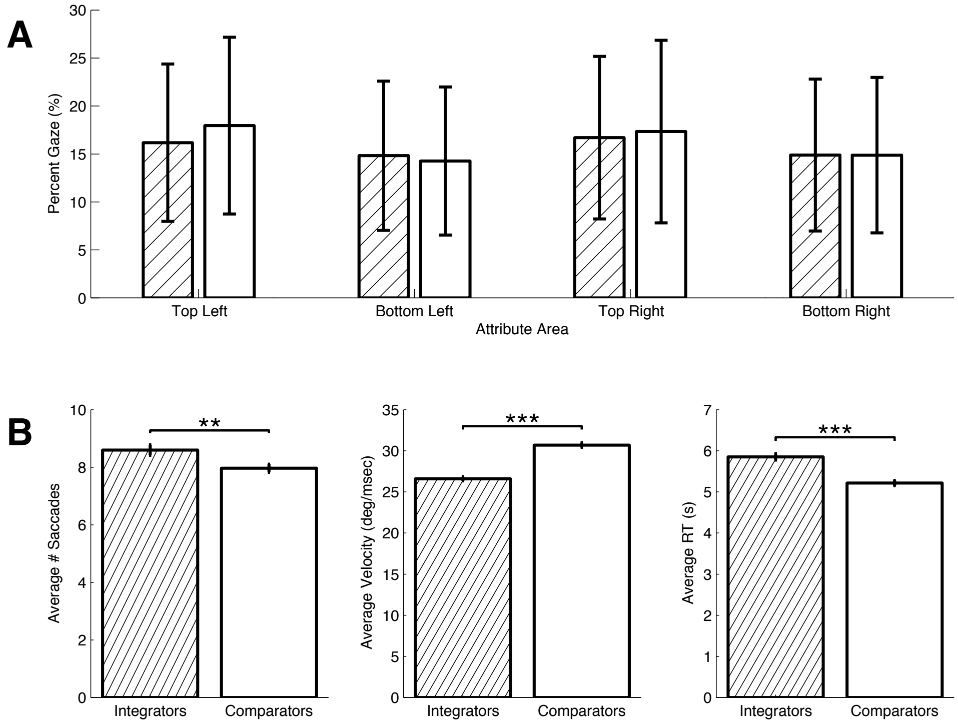


*Figure 1.* (A) Integrators and comparators distribute their gaze in an approximately uniform manner across attribute regions. Error bars denote one standard deviation from their respective means. (B) We additionally tested for other previously studied effects of eye movements. Integrators are associated with a greater number of saccades per trial ($t(5398) = 2.74$, $p = .0061$) and smaller estimated saccade velocities per trial ($t(5257) = -10.46$, $p < .001$); both of these differences likely contribute to the slower response times by comparators, $t(5296) = 6.31$, $p < .001$ (discarding responses slower than 2 standard deviations above the mean). Error bars denote the standard errors of their respective means.

Table 1

*Percentage breakdown of selected transition patterns (only showing non-returning transitions) at the beginning of each trial.*

| Transition Sequence | Occurrences (%) | | |
|---|---|---|---|
| | Population | Integrators | Comparators |
| Top Left - Top Right - Bottom Left - Bottom Right | 10.11 | 10.04 | 10.18 |
| Top Left - Top Right - Bottom Right - Bottom Left | 3.67 | 3.85 | 3.5 |
| Top Left - Bottom Left - Top Right - Bottom Right | 2.15 | 0 | 4.14 |
| Top Left - Bottom Left - Bottom Right - Top Right | 3.02 | 0.23 | 5.61 |
| Top Left - Bottom Right - Top Right - Bottom Right | 0.02 | 0.04 | 0 |
| Top Left - Bottom Right - Bottom Left - Top Right | 0.07 | 0 | 0.14 |
| Top Right - Top Left - Bottom Left - Bottom Right | 0.11 | 0.12 | 0.11 |
| Top Right - Top Left - Bottom Right - Bottom Left | 0.06 | 0.08 | 0.04 |
| Top Right - Bottom Left - Top Left - Bottom Right | 0 | 0 | 0 |
| Top Right - Bottom Left - Bottom Right - Top Left | 0.13 | 0.12 | 0.14 |
| Top Right - Bottom Right - Top Left - Bottom Left | 0.02 | 0.92 | 0.04 |
| Top Right - Bottom Right - Bottom Left - Top Left | 0.04 | 0 | 0.07 |
| Bottom Left - Top Left - Top Right - Bottom Right | 1.02 | 0 | 1.11 |
| Bottom Left - Top Left - Bottom Right - Top Right | 0.02 | 0 | 0.04 |
| Bottom Left - Top Right - Top Left - Bottom Right | 0.02 | 5.54 | 0.04 |
| Bottom Left - Top Right - Bottom Left - Top Left | 0.02 | 0.96 | 0.04 |
| Bottom Left - Bottom Right - Top Left - Top Right | 3.93 | 0.50 | 2.43 |
| Bottom Left - Bottom Right - Top Right - Top Left | 0.94 | 0 | 0.93 |
| Bottom Right - Top Left - Top Right - Bottom Left | 0.61 | 0 | 0.71 |
| Bottom Right - Top Left - Bottom Left - Top Right | 0.02 | 0 | 0.04 |
| Bottom Right - Top Right - Top Left - Bottom Left | 0.04 | 0 | 0.07 |
| Bottom Right - Top Right - Bottom Left - Top Left | 0 | 0 | 0 |
| Bottom Right - Bottom Left - Top Left - Top Right | 0.24 | 0.27 | 0.21 |
| Bottom Right - Bottom Left - Top Right - Top Left | 0.04 | 0.04 | 0.04 |

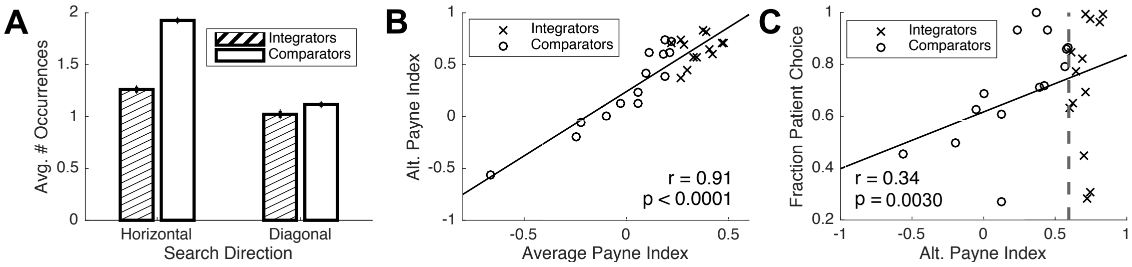


*Figure 2*. (A) The average frequency of diagonal and horizontal transitions (based on tracked gaze coordinates) across sub-populations. Comparators are more likely to perform both horizontal or diagonal transitions relative to integrators. (B) An alternative Payne Index measure not counting diagonal transitions is highly correlated with the measure used throughout the main article. (C) Membership assignment, as well as the main relationship discussed in the paper, is unchanged using a Payne Index not inclusive of diagonal transitions.

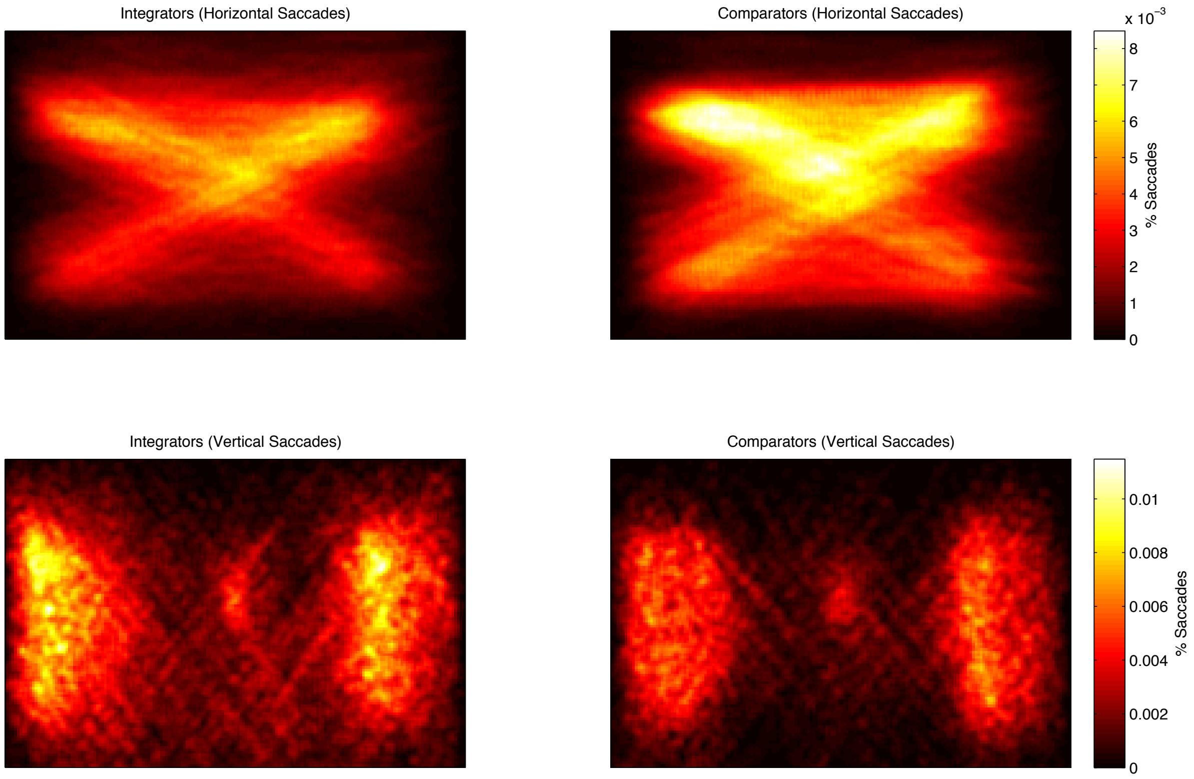


*Figure 3.* Comparators saccades involve a greater occurrence of horizontal movements (top row) while integrators perform more vertically-travelling saccades during the task. Saccade probabilities were computed using a kernel density estimator based on the pooled travel coordinates of all identified saccades.
